# Entwicklung und Validierung einer computerbasierten Aufgabe zur Induktion eines psychischen Beanspruchungsspektrums

**DOI:** 10.1007/s41449-022-00304-y

**Published:** 2022-03-10

**Authors:** Yannick Andreas Funk, Henrike Haase, Julian Remmers, Noé Nussli, Barbara Deml

**Affiliations:** Institut für Arbeitswissenschaft und Betriebsorganisation (ifab), Karlsruher Institut für Technologie, Engler-Bunte-Ring 4, 76131 Karlsruhe, Deutschland

**Keywords:** Psychische Belastung, Psychische Beanspruchung, Rating Scale Mental Effort (RSME), Multiples Ressourcenmodell, Mental workload, Task load, Rating scale mental effort (RSME), Multiple resource theory

## Abstract

Im Rahmen des vom BMBF geförderten Projekts *Fahrerkabine 4.0 *wird eine adaptive Mensch-Maschine-Schnittstelle für Landmaschinen entwickelt, die das aktuelle Beanspruchungslevel mit Hilfe physiologischer Daten detektiert. Zu diesem Zwecke wird in dieser Arbeit eine Experimentalaufgabe entwickelt und evaluiert, die ein psychisches Belastungsspektrum von *wenig* bis *sehr stark anstrengend *in Versuchspersonen induzieren kann. In drei Laborstudien wird psychische Belastung mit einer Überwachungstätigkeit erzeugt, deren Bearbeitungsgeschwindigkeit randomisiert variiert. Die Komplexität der Tätigkeit wird abschnittsweise durch eine visuelle und/oder eine auditive Nebenaufgabe erhöht. Von den Versuchspersonen empfundene psychische Beanspruchung wird mit Hilfe der Rating Scale Mental Effort, der Reaktionszeiten und der Fehlerrate bewertet. Die Studien mit jeweils N = 17, N = 8 bzw. N = 21 Probanden zeigen, dass eine dynamische Kombination von Haupt- und Nebenaufgaben signifikant unterschiedliche Belastungsgrade induzieren kann (*F* (2,40) = 54.834, *p* < 0,001).

*Praktische Relevanz*: Mithilfe der entwickelten Experimentalaufgabe wird in zukünftigen Arbeiten ein Messsystem zur Klassifizierung psychischer Beanspruchungszustände für Landmaschinen entworfen und erprobt. In beanspruchungsarmen Situationen (z. B. automatisierte Ernte) sollen zusätzliche Handlungsempfehlungen vorgeschlagen werden. Während stark beanspruchenden Abschnitten soll eine Überforderung der Nutzenden vermieden werden, indem lediglich die für die Durchführung der Arbeitstätigkeit notwendigen Informationen angezeigt werden.

## Abstract (en)

As part of the driver’s cab 4.0 project funded by the BMBF, an adaptive human-machine interface for agricultural machinery, which detects the current level of mental workload by analysing physiological data is being developed. For this purpose, an experimental task is designed and evaluated, which can induce a mental workload spectrum from little to very strenuous in humans. In three laboratory studies, mental workload is generated by a monitoring activity, with varying difficulty levels. The complexity of the activity is increased by a visual and/or an auditory secondary task. Subjectively perceived mental workload is evaluated by using the Rating Scale Mental Effort, collecting reaction times and error rates. The studies with N = 17, N = 8 and N = 21 participants show that a dynamic combination of main and secondary tasks can induce significantly different degrees of workload (F (2.40) = 54,834, *p* < 0.001).

*Practical Relevance*: The experimental task developed in this paper will be used to design a measuring system for mental workload based on physiological indicators for combine harvesters. In low-workload situations (e.g. automated harvesting) additional recommendations for action should be proposed by the system. During high workload sections excessive demands on the user should be avoided by only showing the information necessary to carry out the task at hand.

## Hintergrund

Der erfolgreiche Einzug intelligenter Mensch-Maschine-Systeme in die Arbeitswelt 4.0 führt zu einem grundlegenden Wandel in der Arbeitsplatzgestaltung: Routinetätigkeiten in allen Wirtschaftssektoren werden teilweise oder vollständig digitalisiert und automatisiert (Eichhorst und Buhlmann 2015). Die Arbeitsaufgaben werden größtenteils komplexer, interaktiver und kreativer. Der bereits begonnene Trend – weg von Routinetätigkeiten und hin zu Nicht-Routinetätigkeiten – wird sich weiter und möglicherweise beschleunigt fortsetzen (Eichhorst und Buhlmann 2015).

Dieser Wandel in der modernen Arbeitswelt führt zu einem Anstieg der psychischen Beanspruchung der Arbeitenden (vgl. z. B. Diebig et al. 2018; Schaff 2019; Poppelreuter und Mierke 2018). Wiederholte Einwirkung hoher psychischer Belastungen kann beispielsweise zu Entfremdungserscheinungen (einer Komponente des Burnout-Syndroms) führen (DIN EN ISO 10075-1:2018 2018). Vor diesem Hintergrund rückt die Entwicklung nutzeradaptiver Schnittstellen zunehmend in den Fokus der Forschung. Ein Ziel personenadaptiver Schnittstellen ist es, den Zustand des Nutzers mit objektiv messbaren und validen Indikatoren zu identifizieren, um gegebenenfalls im Arbeitsprozess Unterstützung oder Zusatzinformationen bereitzustellen (Bornewasser et al. 2018).

Im Rahmen des vom BMBF geförderten Verbundvorhabens „Fahrerkabine 4.0“ (Fahrerkabine 4.0 2019) wird am KIT eine adaptive Mensch-Maschine-Schnittstelle für Landmaschinen entwickelt, die in der Lage ist, das aktuelle Beanspruchungsniveau der Fahrer und Fahrerinnen mit Hilfe physiologischer Daten zu detektieren. Daraus sollen Handlungsempfehlungen abgeleitet werden: so können z. B. bei geringer Beanspruchung während einer automatisierten Erntefahrt zusätzliche Büroaufgaben, die andernfalls am Ende eines langen Tages bearbeitet werden müssen, vorgezogen werden. Bei hoher Beanspruchung, wie etwa bei Wendemanövern, lassen sich unkritische Teilaufgaben oder die Verarbeitung sekundärer Informationen verzögern.

Bei der Entwicklung einer (psychischen) Nutzerzustandserfassung gilt es, zwischen den Begriffen „psychischer Belastung“ und „psychischer Beanspruchung“ zu unterscheiden. Die DIN EN ISO 10075-1:2018 definiert psychische Belastung als „die Gesamtheit aller erfassbaren Einflüsse, die von außen auf einen Menschen zukommen und diesen psychisch beeinflussen“. Psychische Beanspruchung beschreibt die unmittelbare Auswirkung der psychischen Belastung auf das Individuum in Abhängigkeit des aktuellen Zustands. Der Begriff der psychischen Beanspruchung bezieht sich dabei sowohl auf kognitive als auch auf emotionale Vorgänge im arbeitenden Menschen. Diese Prozesse stehen eng miteinander in Beziehung und es ist kaum möglich sie sinnvoll getrennt voneinander zu betrachten (DIN EN ISO 10075-1:2018 2018).

Psychische Beanspruchung beschreibt also die individuelle Reaktion eines Menschen auf alle äußeren, psychischen Einwirkungen (Belastung) und hängt darüber hinaus von den individuellen Fähigkeiten, Fertigkeiten und Eigenschaften der belasteten Menschen ab: Eine gleiche Belastung kann zu individuell unterschiedlicher Beanspruchung führen (Rohmert 1983). Hieraus ergibt sich die Forderung, dass Methoden zur Beanspruchungsmessung die Individualität der Beanspruchungsreaktionen berücksichtigen und speziell für die jeweilige Nutzerin oder den jeweiligen Nutzer kalibriert werden müssen. Zu diesem Fazit kommen auch Jeschke et al. (2016), die den Zusammenhang von psychischer Beanspruchung und physiologischen Indikatoren auf Stichprobenebene – nicht auf individueller Ebene – untersuchten.

Die bisherige Forschung zeigt, dass die adaptive Gestaltung von Unterstützungsmaßnahmen sich positiv auf die menschliche Leistungsfähigkeit und Motivation in der Mensch-Maschine-Interaktion auswirkt und die Effektivität des gesamten Mensch-Maschine-Systems verbessert werden kann (Kyriakidis et al. 2015; Schwarz 2019; Ulahannan et al. 2020; Ramakrishnan et al. 2021). Aktuelle Systeme zur Nutzerzustandserfassung sind größtenteils Totmannschalter, bei denen der Fahrer oder die Fahrerin kontinuierlich ihre Verfügbarkeit signalisieren, z. B. durch Berührung des Lenkrads (Diederichs et al. 2020). Komplexere Systeme, z. B. die Bewertung psychischer Beanspruchung werden bisher nicht in Serienproduktionen verbaut und bedürfen noch weiterer Forschung (Khan und Lee 2019). Ursachen hierfür sind u. a. mangelnde regulatorische Klarheit, die schwankende Zuverlässigkeit der Messsysteme und die Akzeptanz, bzw. das Vertrauen in diese Systeme (Stuiver et al. 2010; Manzey 2012; Khan und Lee 2019; Morales-Alvarez et al. 2020; Pretto et al. 2020; Kalayci et al. 2021).

Zukünftig soll es möglich sein, anhand der in dieser Arbeit entwickelten Experimentalaufgabe psychische Beanspruchungsmesssysteme mit beliebigen physiologischen Indikatoren auf eine individuelle Versuchsperson zu kalibrieren.

Damit ein solches System zunächst im Labor entwickelt und anschließend an einem Demonstrator erprobt werden kann, wird eine Experimentalaufgabe konzipiert, mit deren Hilfe sich ein Spektrum psychischer Beanspruchungszustände reproduzierbar in Versuchspersonen induzieren lässt. Auf dieser Arbeit aufbauend können in Zukunft verschiedene Messsysteme (EKG, Eye Tracker, Stimmfrequenzanalyse, etc.), im Rahmen diverser Forschungsprojekte und Abschlussarbeiten, im Hinblick auf ihre Eignung zur psychischen Beanspruchungsanalyse in einem validierten Umfeld untersucht werden.

### Erfassung psychischer Beanspruchung

Die Quantifizierung psychischer Beanspruchung erfolgt in drei Kategorien: subjektiv erlebt, physiologisch gemessen und leistungsbasiert. Die Erhebung subjektiv erlebter psychischer Beanspruchung geschieht mit Hilfe von Ratingskalen, Fragebögen, Checklisten oder Interviews. Dem liegt die Annahme zugrunde, dass das Beanspruchungsempfinden von Individuen auf eine tatsächliche äußere Belastung zurückzuführen ist und diese von den Versuchspersonen differenziert wiedergegeben werden kann (Schlick et al. 2018). Psychophysiologische Messungen (z. B. Herzschlagfrequenz und Augenbewegungen) gelten – im Gegensatz zur subjektiven Befragung – als objektiv, da die Versuchspersonen diese für gewöhnlich nicht bewusst beeinflussen können. Ein weiterer Vorteil physiologischer Messungen ist die kontinuierliche Datenerfassung, wobei die Arbeitstätigkeit nicht durch Befragungen unterbrochen werden muss (Schlick et al. 2018; Jeschke et al. 2016).

De Waard (1996) identifizierte einen *n*-förmigen Zusammenhang zwischen Leistung und psychischer Beanspruchung. Demnach führen sowohl eine sehr geringe Beanspruchung (Monotonie) als auch eine sehr große Beanspruchung (Überforderung) zu einem Leistungsabfall. Die Leistung einer Person hängt dabei nicht nur von ihrer individuellen Kapazität, sondern auch von ihrem Willen ab, die Aufgabe zu lösen. Diese Bereitschaft, eine Aufgabe zu lösen, kann sich während einer Tätigkeit mehrfach ändern. So kann eine Versuchsperson Ermüdung und steigende Aufgabenschwierigkeit zu einem gewissen Grad durch Willenskraft kompensieren. Die psychische Beanspruchung kann also variieren, während die gemessene Leistung gleichbleibt.

Die Erforschung psychischer Beanspruchung und deren diagnostisches Potenzial für adaptive Mensch-Maschine-Interaktion sind nicht neu. Rouse (1988) beispielsweise entwickelte das Konzept des *Adaptive Aiding* für den Bereich der Flugzeugführung. Dabei sollten der Pilot oder die Pilotin nur dann durch Automation unterstützt werden, wenn diese Unterstützung auch tatsächlich zur Aufgabenerfüllung notwendig war. Als Indikatoren für Unterstützungsbedarf wurden Leistungsparameter (z. B. Änderung der Reaktionszeit) verwendet. De Waards (1996) Ergebnisse legen jedoch nahe, dass die Messung psychischer Beanspruchung nicht allein auf Leistungsparametern beruhen kann, sondern durch physiologische Maße ergänzt werden sollte. Diese Erkenntnis wurde in spätere Forschungsarbeiten einbezogen, woraufhin die Leistungsmessung um physiologische Indikatoren ergänzt wurde. Weitere Arbeiten zur Entwicklung adaptiver Schnittstellen sind u. a. Morrison et al. (2006), Stanney et al. (2009), Schneider (2019), Schwarz (2019), Luong et al. (2020), Planke et al. (2021) und Hillege et al. (2020). Eine Übersicht weit verbreiteter physiologischer Messverfahren findet sich beispielsweise bei Hancock et al. (2021).

### Kognitive Informationsverarbeitung

**Figure 1:**
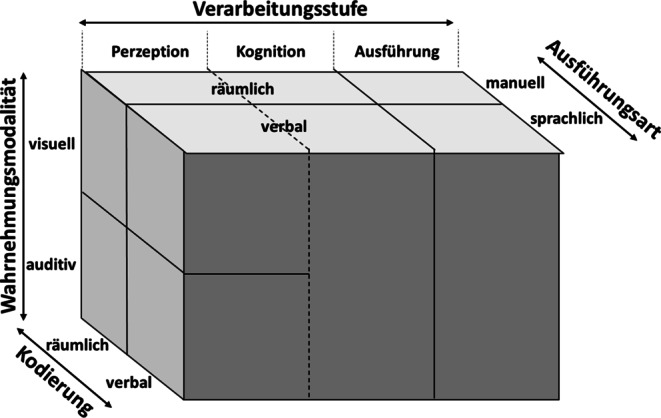

Wickens (1984, 2002, 2008) *Multiple Resource Theory *untersucht unterschiedliche Ressourcendimensionen mit Bezug zu psychischer Beanspruchung und stellt diese grafisch in einem Würfelmodell dar (vgl. Abb. 1). Die kognitive Informationsverarbeitung wird in die drei Stufen *Perzeption* (Gesamtheit der Wahrnehmung), *Kognition* (Verarbeitung der wahrgenommenen Informationen) und *Ausführung* (Reaktion auf wahrgenommene Information) eingeteilt. Wickens (2002) konnte durch Untersuchungen an *Dual-Task-Situationen* zeigen, dass perzeptive und kognitive Prozesse dieselbe Ressource beanspruchen, während die Ausführung einer Handlung auf andere Ressourcen zurückgreift. Die parallele Ausführung einer perzeptiven und kognitiven Aufgabe, z. B. eine visuelle Überwachungsaufgabe (Perzeption) gekoppelt mit einer mentalen Rotation (Kognition), führt also zu Interferenzen und damit zu hoher psychischer Beanspruchung und gegebenenfalls zu Leistungseinbußen.

Neben den Verarbeitungsstufen unterscheidet Wickens (1984, 2002, 2008) die *räumliche* und *verbale* Kodierung von Informationen sowie die Wahrnehmungsmodalitäten *visuell* und *auditiv.* Intramodale Dual-Task-Situationen wie das Lesen eines Textes (visuell) während des Autofahrens (hohe visuelle Anforderungen) führen zu Interferenz und damit zu höherer psychischer Beanspruchung als übermodale Situationen wie das Hören eines Textes (auditiv) während des Autofahrens. Analog beeinflusst die Kodierung einer Information die erlebte psychische Beanspruchung: übermodale Informationsverarbeitung führt zu Interferenz, falls die Information in beiden Fällen gleich kodiert ist. So fällt es schwer, Nachrichten zu hören (verbal, auditiv) und gleichzeitig einen Text zu lesen (verbal, visuell).

Zusammenfassend bedeutet dies für die Gestaltung der Experimentalaufgabe, dass die empfundene psychische Beanspruchung einer (Haupt‑) Aufgabe durch Hinzufügen einer oder mehrerer paralleler (Neben‑) Aufgaben gesteigert werden kann. Der Grad der gesteigerten Beanspruchung richtet sich dabei danach, ob die angesprochenen Ressourcendimensionen bei der Kombination miteinander interferieren oder, wie im Falle einer übermodalen Situation mit unterschiedlicher Kodierung, unterschiedliche Ressourcen beanspruchen.

## Bausteine der Experimentalaufgabe

In der Arbeitswissenschaft wird psychische Beanspruchung oft über sog. Dual-Task-Paradigmen induziert und die physiologischen Reaktionen darauf gemessen (z. B. Matthews et al. 2015; Faure et al. 2016; Solís-Marcos und Kircher 2019; Moacdieh et al. 2020; Zokaei et al. 2020). In diesen Studien wird die Reliabilität verschiedener Leistungsindikatoren und physiologischer Parameter zur Diagnose psychischer Beanspruchung untersucht, indem Versuchspersonen Aufgaben bearbeiten, die phasenweise um Nebenaufgaben ergänzt werden. Parallel werden Leistungsmaße, subjektive Beanspruchung (z. B. NASA TLX oder RSME) und physiologische Parameter erhoben. Oft wird die Eignung eines speziellen Messverfahrens oder Indikators zur Beanspruchungsmessung untersucht (z. B. Moacdieh et al. 2020). Die unterschiedlichen Rahmenbedingungen der einzelnen Studien erschweren den direkten Vergleich der angewandten Messgeräte und -verfahren untereinander.

Ziel der hier vorgestellten Studie ist es, eine standardisierte Aufgabe (bestehend aus gekoppelten Teilaufgaben) zu entwickeln, die nachweislich in der Lage ist, variierende psychische Beanspruchung in Versuchspersonen zu induzieren, um anschließend systematisch verschiedene Messgeräte zu untersuchen und diese direkt miteinander zu vergleichen. Die entwickelte Aufgabe kann weiterhin zum Sammeln von Trainingsdaten für maschinelles Lernen und somit als Kalibrierungsaufgabe für Beanspruchungsmesssysteme angewandt werden.

Auf Wickens (2002) *Multiple *Resource Theory aufbauend (vgl. Abschn. 1.2), wurde ein Aufgabenkonzept erarbeitet, dass in drei sukzessiven Laborstudien evaluiert wurde. Dabei wird zwischen einer Hauptaufgabe (HA), einer visuellen und einer auditiven Nebenaufgabe (NA) unterschieden.

Bei der Hauptaufgabe handelt es sich um eine bildschirmbasierte Überwachungstätigkeit in fünf Geschwindigkeitsstufen. Auf einem Computermonitor (1920 × 1080 Pixel, 22 Zoll) wird ein Video von einer Mähdrescherfahrt aus der Egoperspektive gezeigt. Dieses Video ist generisch und kann für beliebige Überwachungssituationen wie z. B. Leitwarten oder Luftüberwachung angepasst werden. Während des Videos werden randomisiert die Buchstaben „W“, „A“ oder „D“ in fünf Frequenzstufen auf dem Bildschirm eingeblendet (alle 15, 10, 5, 3 oder 2 s, im Folgenden als Stufe 1–5 bezeichnet). Die Aufgabe der Versuchspersonen besteht darin, die angezeigten Buchstaben auf einer Computertastatur mit der linken Hand zu drücken.

Die auditive Nebenaufgabe besteht aus zehn Mal 13 Fragen, die den Versuchspersonen parallel zur Hauptaufgabe über Kopfhörer präsentiert werden. Ein Block aus 13 Fragen dauert zwei Minuten, inklusive Antworten. Die Antworten der Probanden und Probandinnen werden dabei über ein externes USB-Mikrofon aufgezeichnet. Der Fragenkatalog baut auf den Arbeiten von Batliner et al. (2006), Fernandez und Picard (2003), Scherer et al. (2008) und Wittels et al. (2002) auf und wurde unter zwei Hauptkriterien entwickelt: (1) Jeder 13-Fragen-Block sollte denselben Gesamtschwierigkeitsgrad aufweisen, um eine konstante Belastung der Versuchspersonen zu realisieren. (2) Für eine effektive Analyse verschiedener Stimmparameter (z. B. Frequenz, Jitter, Tonhöhe; vgl. Sharma und Gedeon 2012) sollten sich einzelne Wörter und Silben in den Antworten verschiedener Blöcke wiederholen. Die einzelnen Fragen werden in vier Schwierigkeitsklassen eingeteilt:

1. Sehr gering: Wiederholung einfacher, kurzer Sätze und lautes Hochzählen von eins bis sieben, acht, neun oder zehn.
2. Gering: Wiederholung zusammenhangsloser Silben und „Zungenbrecher“.
3. Mittel: Einfache mathematische Operationen (z. B. „40 + 12 = ?“) und übersetzen einfacher Sätze aus dem Englischen.
4. Hoch: Fragen zum Allgemeinwissen, z. B. nach Hauptstädten Europas oder Ozeanen.

Jeder 13-Fragen-Block enthält drei Fragen sehr geringer, vier Fragen geringer, vier Fragen mittlerer und zwei Fragen hoher Schwierigkeit. Um Reihenfolgeeffekte (vgl. z. B. Döring und Bortz 2016) zu vermeiden, werden die Fragen innerhalb der Blöcke und die Blöcke selbst randomisiert präsentiert.

Nach Wickens (2002) handelt es sich bei der Kombination *Überwachung* *+* *auditive Nebenaufgabe* um eine übermodale Dual-Task-Situation (visuell & auditiv) unterschiedlicher Ausführungsarten (manuell & sprachlich) und Kodierung (räumlich & verbal). Mehrfachbelegung derselben Ressource (Interferenzerscheinungen) werden nur bei Fragen der dritten und vierten Schwierigkeitsklasse erwartet, bei denen die Perzeption (Überwachung) durch zusätzliche Kognition (Finden der korrekten Antwort) angegriffen wird.

**Figure 2:**
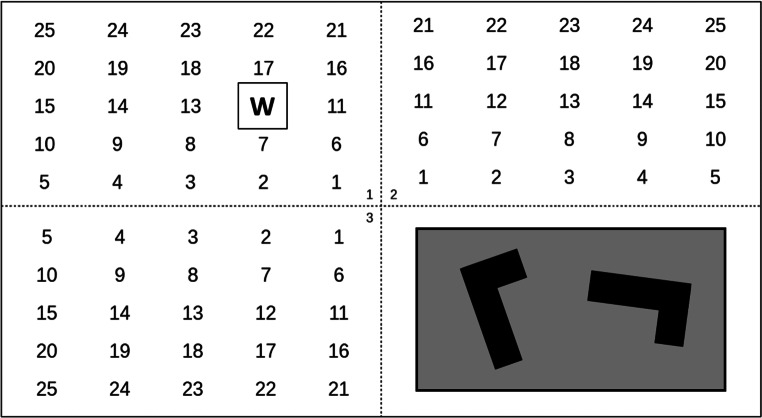

Bei der visuellen Nebenaufgabe handelt es sich um eine mentale Rotation (Eggemeier und Wilson 1991). Dabei werden im unteren rechten Bildschirmquadranten vier verschiedene Figurenpaare in einem Abstand von drei Sekunden randomisiert anzeigt. Die Figuren sind entweder zueinander gedreht (randomisierter Winkel) oder zueinander gedreht und zusätzlich gespiegelt. Die Aufgabe der Versuchspersonen besteht darin, zusätzlich zur Hauptaufgabe, mit der rechten Hand anzuzeigen, ob es sich bei der mentalen Rotation um eine *Drehung* (linke Pfeiltaste) oder um eine *Drehung mit Spiegelung* (rechte Pfeiltaste) handelt (vgl. Abb. 2).

Nach Wickens (2002) handelt es sich bei dieser Dual-Task-Situation *Überwachung* *+* *Mentale Rotation* um eine intramodale (visuelle) Kombination mit Bezug zu allen Verarbeitungsstufen (Überwachungsaufgabe = Perzeption, Mentale Rotation = Kognition, und Betätigen der Tastatur = Ausführung). Die Kodierung der Hauptaufgabe erfolgt sowohl räumlich (schnelles Erkennen randomisierter Buchstabenpositionen) als auch verbal (richtiges Zuordnen der präsentierten Buchstaben). Die Kodierung der visuellen Nebenaufgabe ist ausschließlich räumlich (kognitive Kongruenzprüfung). Wickens (2002) Modell folgend wird bei dieser Aufgabenkombination deutlich mehr Interferenz innerhalb der Ressourcendimensionen hervorgerufen als durch die auditive Nebenaufgabe. Folglich wird auch eine höhere psychische Beanspruchung erwartet.

Der höchste Grad psychischer Beanspruchung wird bei der Kombination aller Aufgabenteile (Hauptaufgabe + auditive + visuelle Nebenaufgabe) erwartet. Diese Triple-Task-Situation belegt alle Ressourcendimensionen der *Multiple Resource Theory* und führt zu erheblichen Interferenzen innerhalb dieser Dimensionen.

Zusammenfassend werden folgende Hypothesen festgehalten:

1. Die induzierte psychische Beanspruchung der Hauptaufgabe steigt bei Kürzung der Buchstabenfrequenz an.
2. Die erlebte psychische Beanspruchung der Versuchspersonen kann durch Hinzufügen von Nebenaufgaben gesteigert werden.
3. Die geringste Steigerung wird durch die auditive Nebenaufgabe, eine höhere durch die visuelle Nebenaufgabe und eine sehr große durch die Kombination von visueller und auditiver Nebenaufgabe induziert.

Die zu entwickelnde Experimentalaufgabe soll Trainingsdaten für die mathematische Modellbildung generieren. Ein Trainingsdatensatz besteht dabei aus zwei Teilen, den Prädiktoren und der Antwortvariable (vgl. z. B. McCullagh und Nelder 2019). Bei den Prädiktoren handelt es sich in unserem Fall um physiologische Messdaten, die mit beliebigen Systemen erhoben werden können.

Psychische Beanspruchung ist umso besser messbar, je präziser die Antwortvariable des Trainingsdatensatzes sie repräsentiert. Für die zu entwickelnde Aufgabe fiel die Wahl auf die vielfach erprobte und etablierte *Rating Scale Mental Effort* (RSME; Zijlstra 1993), bei der die Bewertung der subjektiven Beanspruchung auf einer Skala von 0 bis 150 erfolgt. Als Orientierung dienen auf der Skala neun verbal kodifizierte Ankerpunkte von „gar nicht anstrengend“ bis „außerordentlich anstrengend“. Als Einskalen-Fragebogen bietet die RSME ein wenig invasives Messinstrument und liefert vergleichbare Beanspruchungseinschätzungen zum NASA TLX (Ghanbary Sartang et al. 2016; Longo und Orrú 2020).

Die RSME wird in dieser Arbeit zweifach angewendet: Im ersten Schritt (vgl. Abschn. 4.1) bewerten die Versuchspersonen die induzierte psychische Beanspruchung einzelner, randomisierter Aufgabenkombinationen. Im zweiten Schritt (vgl. Abschn. 5.2) wird anhand dieser Bewertung eine feste Aufgabenreihenfolge definiert, die als fertige Experimentalaufgabe zum Einsatz kommt. Mithilfe der so entwickelten Experimentalaufgabe werden zukünftig physiologische Messdaten und RSME-Bewertungen gesammelt, um individuelle, an Versuchspersonen angepasste Beanspruchungsmodelle zu errechnen.

Alle Aufgaben sind in Python (2021) implementiert und lassen sich beliebig kombinieren.

## Laborstudien

Zwischen Juli 2020 und Mai 2021 wurden insgesamt drei Laborstudien zur Evaluierung von Kombinationen aus Haupt- und Nebenaufgaben durchgeführt. In Studie 1 wurden RSME-Bewertungen und Leistungsdaten für die Hauptaufgabe ohne Nebenaufgaben erhoben. Studie 2 diente als Vorstudie zu Studie 3. Dabei wurde der Einfluss der visuellen Nebenaufgabe untersucht, um die Validität des Gesamtvorhabens abzuschätzen. In Studie 3 wurden schließlich drei verschiedene Kombinationen aus Haupt- und Nebenaufgabe evaluiert.

### Stichprobe

Das Probandenkollektiv umfasste 17 Personen (11 Frauen, 6 Männer) im Alter von 22 bis 42 Jahren (M = 26,5, SD = 4,55) in Studie 1, 8 Personen (3 Frauen, 5 Männer) im Alter von 23 bis 29 Jahren (M = 26,71, SD = 2,85) für Studie 2 und 21 Personen (9 Frauen, 12 Männer) im Alter von 20 bis 30 Jahren (M = 23,6, SD = 2,89) in Studie 3. Alle Stichproben wurden aus freiwilligen Studierenden und Mitarbeitenden des KIT rekrutiert. Die Studien wurden durch die Ethikkommission des KIT genehmigt. Alle Versuchspersonen wurden vorab über den Untersuchungsablauf, ihre Rechte und die Anonymität der Daten informiert. Einverständniserklärungen liegen vor. Es wurde darauf geachtet, dass keine Einschränkungen des Sehvermögens vorlagen bzw. dass geeignete Sehhilfen für die Experimente benutzt wurden.

### Versuchsablauf

**Figure 3:**
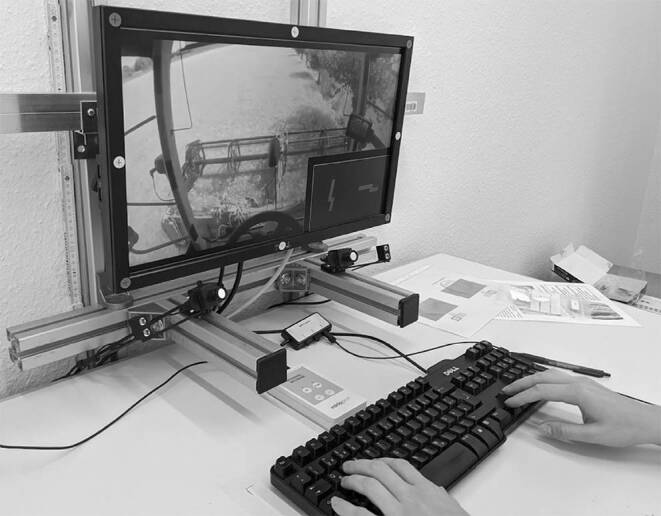

Die Studien begannen mit der Instruktion der Versuchspersonen. Die Probanden und Probandinnen wurden gebeten, eine Versuchsbeschreibung, eine Datenschutzerklärung und eine Einverständniserklärung zu lesen und zu unterschreiben. Nach Erhebung der versuchsrelevanten demografischen Daten (Geschlecht, Alter, Sehhilfen) und der Beantwortung von Fragen nahmen die Versuchspersonen vor einem Bildschirm Platz, um das Experiment durch Drücken der Leertaste selbständig zu beginnen (vgl. Abb. 3). Bei allen drei Studien wurden die Teilnehmenden angewiesen, eine korrekte Antwort einer möglichst schnellen Antwort vorzuziehen.

Die Erhebung von Studie 1 fand zwischen Juli und August 2020 statt und galt der Evaluierung der Hauptaufgabe ohne Nebenaufgaben (vgl. Abschn. 2). Jede Frequenzstufe wurde zwei Minuten lang anzeigt, gefolgt von 15 s Pause ohne Anzeige weiterer Buchstaben. In diesem Zeitfenster bewerteten die Versuchspersonen ihre subjektiv empfundene psychische Beanspruchung auf einer Skala von 0 bis 150 (RMSE). Das Experiment begann und endete mit einer 60-sekündigen Baseline, während der das Video weiterlief, aber keine Buchstaben angezeigt wurden. Jeder Proband und jede Probandin absolvierte zwei Durchläufe, unterbrochen durch eine kurze Pause. Der erste Durchgang wurde als Übungsphase gewertet. Die gesamte Versuchsdurchführung dauerte 29 min exklusive Anleitung.

Studie 2 lief im September 2020 in Vorbereitung zu Studie 3. Studie 2 erweiterte Studie 1 bei identischer Hauptaufgabe um die Einblendung einer visuellen Nebenaufgabe in Form einer mentalen Rotation im unteren rechten Bildschirm-Quadranten.

Studie 3 fand zwischen Februar und April 2021 statt. In dieser Studie wurde die Versuchsdurchführung um eine Übungsphase ergänzt, in der drei Aufgabenkombinationen separat bearbeitet werden. Die Übungszeit für die Hauptaufgabe und für die visuelle Nebenaufgabe betrug jeweils zwei Minuten. Die Übung zur auditiven Nebenaufgabe bestand aus drei Fragen, die über Kopfhörer präsentiert wurden. Nach der Übungsphase wurden in randomisierter Reihenfolge drei Aufgabenkombinationen präsentiert: *HA* *+* *visuelle NA, HA* *+* *auditive NA, HA* *+* *auditive NA* *+* *visuelle NA*.

Analog den zu Studien 1 und 2 wurde auch jede Stufe der Studie 3 zwei Minuten lang bearbeitet. Nach jeder Stufe hatten die Versuchspersonen 15 s Zeit zur Abgabe ihrer RSME-Bewertung. Zwischen den Aufgabenkombinationen gab es eine kurze Pause, und jede Kombination begann und endete mit einer 60-sekündigen Baseline, während der das Video ohne Anzeige von Haupt- oder Nebenaufgaben weiterlief. Ein Versuch dauerte 46 min exklusive Anleitung.

Um Reihenfolgeeffekte (vgl. z. B. Döring und Bortz 2016) bei der Präsentation der Stufen und der Aufgabenkombinationen zu vermeiden, wurden sowohl die Stufen der Hauptaufgabe (Studie 1, 2 und 3) innerhalb der Aufgabenkombinationen, als auch die Reihenfolge der Kombinationen (Studie 3) randomisiert. Nach der Instruktion der Versuchspersonen sowie der Beantwortung von Fragen, verließ die Versuchsleitung den Raum und verfolgte den Ablauf auf duplizierten Monitoren in einem angrenzenden Laborraum. Damit sollten zum einen das Übertragungsrisiko von Covid-19 Infektionen und zum anderen potenzielle Versuchsleiter-Effekte (z. B. Erwartungs- oder Pygmalion-Effekt, vgl. Städtler 1998) verringert werden.

### Statistische Auswertung

Die aufgenommenen Daten wurden mit der Software IBM Statistics 26 (IBM 2021) und Matlab 2021a (Mathworks 2021) ausgewertet. Die Studien 1 und 3 erfüllen, falls nicht anders beschrieben, die Anforderungen an parametrische Auswerteverfahren (Field 2013): Intervallskalierung und Normalverteilung der abhängigen Variable. Für messwiederholte (ein- oder zweifaktorielle) ANOVAs wurde zusätzlich auf Sphärizität (vgl. Mauchly 1940) und bei konventionellen ANOVAs auf Varianzhomogenität (Levene’s Test; vgl. Field 2013) geprüft. Sollte die Annahme der Sphärizität verletzt sein, wird eine Greenhouse-Geisser Korrektur angewandt.

Aufgrund von Varianzheterogenität wurde bei der der Analyse der Fehler der Hauptaufgabe (Abschn. 4.2.4) eine Welch-ANOVA anstatt einer konventionellen ANOVA berechnet (Welch 1947).

Bei der Analyse der Fehler der auditiven Nebenaufgabe (Abschn. 4.2.6) wird trotz einer Verletzung der Annahme der Normalverteilung eine messwiederholte ANOVA berechnet. Schminder et al. (2010) untersuchten den Einfluss der Verletzung der Normalverteilungsannahme auf die konventionelle ANOVA und Oberfeld und Franke (2013) auf die messwiederholte ANOVA. Beide Berichte kommen zu dem Schluss, dass die ANOVA robust gegenüber einer Verletzung der Normalverteilungsannahme ist.

Post-Hoc paarweise Vergleiche wurden mit der konservativen Bonferroni-Korrektur durchgeführt. Die Berechnung der Effektstärke erfolgte über das partielle Eta-Quadrat, und einer Bewertung nach Cohen (1988) mit geringen (0,1–0,3), mittleren (0,3–0,5) und stärkeren (>0,5) Effekten. Die Ergebnisse aus Studie 2 wurden auf Grund der geringen Stichprobengröße und ihrer Einstufung als Vorstudie nicht statistisch analysiert.

## Ergebnisse

In diesem Abschnitt werden die Ergebnisse der statistischen Analyse der subjektiven Beanspruchungsbewertung (RSME) und der Leistungsdaten (Reaktionszeit und Fehlerrate) dargestellt. Die Leistungsdaten werden dabei getrennt nach Aufgabentyp betrachtet (Hauptaufgabe, visuelle oder auditive Nebenaufgabe).

### Subjektive Beanspruchung (RSME)

**Figure 4:**
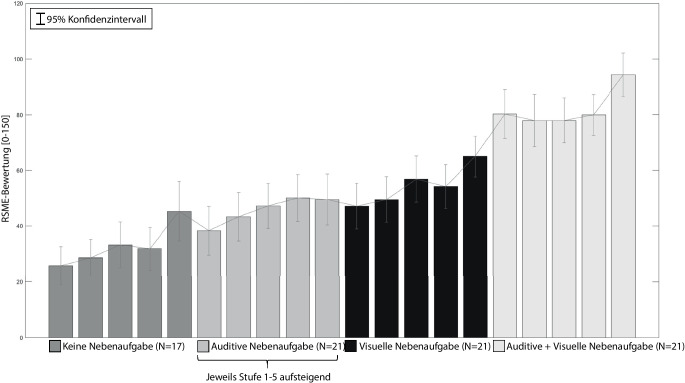

Tab. 1 zeigt einen Überblick der gemittelten subjektiven Beanspruchung der Studien 1, 2 und 3. Die deskriptiven Daten lassen bereits einen Trend im Hinblick auf die zu überprüfenden Hypothesen (vgl. Abschn. 2) erkennen: Die Erhöhung der Buchstabenfrequenz (Hypothese 1), das Hinzufügen von Nebenaufgaben (Hypothese 2) und deren Kombinationen (Hypothese 3) führen zu einem quasi-monoton steigenden Verlauf der psychischen Beanspruchung (vgl. Abb. 4).

**Table 1:** 

	*N*	Nebenaufgabe	Stufe	M (RSME)	SD (RSME)
Studie 1	17	Keine	Stufe 1	25,76	14,22
			Stufe 2	28,59	13,71
			Stufe 3	33,24	17,19
			Stufe 4	31,82	16,22
			Stufe 5	45,29	22,49
Studie 2	8	Visuell	Stufe 1	56,00	16,19
			Stufe 2	66,00	22,48
			Stufe 3	67,14	14,96
			Stufe 4	75,00	19,37
			Stufe 5	79,29	14,27
Studie 3	21	Auditiv	Stufe 1	38,29	20,54
			Stufe 2	43,62	20,47
			Stufe 3	47,19	18,95
			Stufe 4	50,10	19,66
			Stufe 5	49,52	21,41
		Visuell	Stufe 1	47,14	19,30
			Stufe 2	49,52	19,03
			Stufe 3	56,86	19,31
			Stufe 4	54,14	18,45
			Stufe 5	65,00	17,25
		Auditiv + visuell	Stufe 1	80,29	20,76
			Stufe 2	77,95	22,08
			Stufe 3	78,00	18,93
			Stufe 4	80,00	17,06
			Stufe 5	94,05	18,47

Eine einfaktorielle Varianzanalyse (ANOVA) mit Messwiederholung und Greenhouse-Geisser Korrektur ergab, dass sich die RSME-Bewertung der fünf Stufen in Studie 1 statistisch signifikant mit geringer Effektstärke unterscheiden (*F* (1,96, 31,35) = 6,092, *p* = 0,006, partielles η^2^ = 0,276). Mit einem Bonferroni Post-Hoc-Test waren keine signifikanten Unterschiede zwischen den einzelnen Stufen festzustellen.

Eine zweifaktorielle ANOVA mit Messwiederholung mit den Ergebnissen aus Studie 3 zeigte, dass sich die RSME-Bewertung sowohl für die Aufgabenkombinationen mit starkem Effekt (*F* (2,40) = 54,834, *p* < 0,001, partielles η^2^ = 0,733), als auch für die Frequenzstufen mit mittlerem Effekt (*F* (4,80) = 18,265, *p* < 0,001, partielles η^2^ = 0,477) signifikant unterscheiden. Zwischen den Kombinationen und den Frequenzstufen bestehen geringe Interaktionseffekte (*F* (8,160) = 2,754, *p* = 0,007, partielles η^2^ = 0,121).

Die Untersuchung der Interaktionseffekte durch Sichtung der Profildiagramme ergab eine hybride Interaktion. Die Randmittel des Faktors „Aufgabenkombination“ verlaufen mit ähnlichem Trend, ohne Überschneidungen. Die Linienzüge des Faktors „Stufen“ überschneiden sich für die Stufen 4 & 3 und 1 & 2. Daraus folgt, dass der Haupteffekt der Aufgabenkombination uneingeschränkt und der Haupteffekt der Stufen nur unter Vorbehalt interpretiert werden können (vgl. Field 2013). Tab. 2 stellt die Ergebnisse eines Bonferroni-Post-Hoc-Tests der Aufgabenkombinationen dar. Tab. 3 enthält alle signifikanten Ergebnisse der Stufen über die Kombinationen hinweg.

**Table 2:** 

Sample 1	Sample 2	Mittelwertdifferenz	Sig.
Visuelle NA	Auditive NA	±8,790	0,070
Visuelle NA	Visuelle + Auditive NA	±27,524	<0,001
Auditive NA	Visuelle + Auditive NA	±36,314	<0,001

**Table 3:** 

Sample 1	Sample 2	Mittelwertdifferenz	Sig.
Stufe 1	Stufe 3	±5,444	0,027
Stufe 1	Stufe 4	±6,175	0,021
Stufe 1	Stufe 5	±14,286	<0,001
Stufe 2	Stufe 5	±12,492	<0,001
Stufe 3	Stufe 5	±8,841	<0,001
Stufe 4	Stufe 5	±8,111	<0,001

### Leistungsdaten

#### Reaktionszeit der Hauptaufgabe

**Figure 5:**
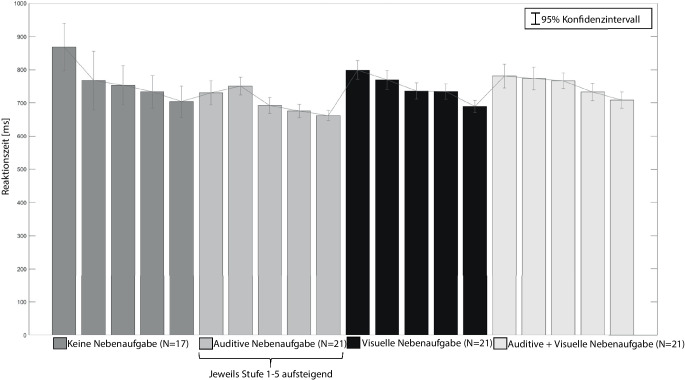

Die Mittelwerte der Reaktionszeiten der Hauptaufgabe sind in Abb. 5 dargestellt.

Eine einfaktorielle ANOVA mit Messwiederholung und Greenhouse-Geisser Korrektur über die mittleren Reaktionszeiten je Stufe aus Studie 1 zeigte signifikante Unterschiede mit mittlerer Effektstärke (*F* (1,84, 29,50) = 8,030, *p* = 0,002, partielles η^2^ = 0,334). Signifikante Ergebnisse des Bonferroni-Post-Hoc-Vergleichs sind in Tab. 4 dargestellt.

**Table 4:** 

Sample 1	Sample 2	Mittelwertdifferenz	Sig.
Stufe 1	Stufe 3	±115,673 [ms]	<0,001
Stufe 1	Stufe 4	±135,238 [ms]	0,003
Stufe 1	Stufe 5	±165,603 [ms]	<0,001

Die Reaktionszeiten in Studie 3 wurden mit einer zweifaktoriellen ANOVA mit Messwiederholung und Greenhouse-Geisser Korrektur untersucht. Die Ergebnisse zeigen, dass sich die Reaktionszeit sowohl für die Kombinationen mit starkem Effekt (*F* (2,34) = 21,984, *p* < 0,001, partielles η^2^ = 0,564) als auch für die Stufen mit starkem Effekt (*F* (2,643, 27,133) = 28,524, *p* < 0,001, partielles η^2^ = 0,627) signifikant unterscheiden. Zwischen den Kombinationen und den Frequenzstufen bestehen keine signifikanten Interaktionseffekte (*F* (4,935, 83,894) = 1,843, *p* = 0,114, partielles η^2^ = 0,098). Alle signifikanten Ergebnisse eines Bonferroni-Post-Hoc-Tests der Aufgabenkombinationen und der Stufen sind in den Tab. 5 und 6 dargestellt.

**Table 5:** 

Sample 1	Sample 2	Mittelwertdifferenz	Sig.
Auditive NA	Visuelle NA	±43 [ms]	<0,001
Auditive NA	Auditive + Visuelle NA	±51 [ms]	<0,001

**Table 6:** 

Sample 1	Sample 2	Mittelwertdifferenz	Sig.
Stufe 1	Stufe 3	±42 [ms]	0,011
Stufe 1	Stufe 4	±55 [ms]	<0,001
Stufe 1	Stufe 5	±86 [ms]	<0,001
Stufe 2	Stufe 4	±44 [ms]	<0,001
Stufe 2	Stufe 5	±74 [ms]	<0,001
Stufe 3	Stufe 5	±44 [ms]	<0,001
Stufe 4	Stufe 5	±30 [ms]	0,010

#### Reaktionszeit der visuellen Nebenaufgabe

Die Reaktionszeit für die visuelle Nebenaufgabe kann nur für zwei Kombinationen – *visuelle NA* *+* *HA* und *visuelle NA* *+* *auditive NA* *+* *HA* – in Studie 3 erhoben werden. Eine zweifaktorielle ANOVA mit Messwiederholung und Greenhouse-Geisser Korrektur ergab keine signifikanten Unterschiede in den Reaktionszeiten, weder zwischen den Kombinationen (*F* (1,19) = 0,07, *p* = 0,794, partielles η^2^ = 0,004), noch zwischen den Stufen (*F* (4,76) = 0,756, *p* = 0,557, partielles η^2^ = 0,038).

#### Reaktionszeit der auditiven Nebenaufgabe

**Figure 6:**
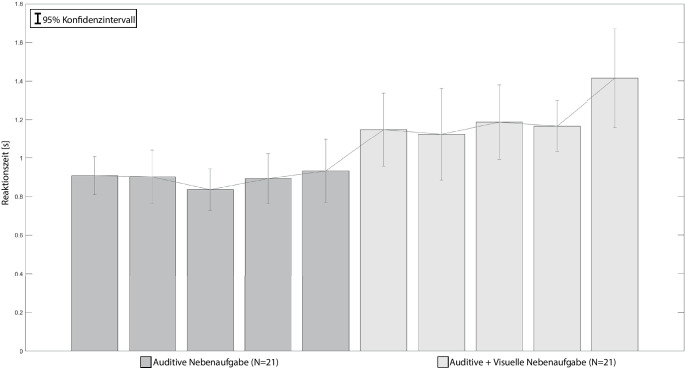

Die Mittelwerte der Reaktionszeiten der auditiven Nebenaufgabe sind in Abb. 6 dargestellt. Analog zu Abschn. 4.2.2 wurde die auditive Nebenaufgabe in zwei von drei Kombinationen in Studie 3 präsentiert. Eine zweifaktorielle ANOVA mit Messwiederholung und Greenhouse-Geisser Korrektur über die Reaktionszeiten ergab signifikante Haupteffekte sowohl zwischen den Kombinationen mit starkem Effekt (*F* (1,20) = 23,518, *p* < 0,001, partielles η^2^ = 0,540) als auch zwischen den Stufen mit geringem Effekt (*F* (2,718, 54,361) = 3,455, *p* = 0,026, partielles η^2^ = 0,147). Zwischen den Kombinationen und den Frequenzstufen bestehen geringe Interaktionseffekte (*F* (2,581, 51.630) = 3,349, *p* = 0,032, partielles η^2^ = 0,143). Die Sichtung der Profildiagramme (vgl. Field 2013) zeigt, dass zwischen dem Faktor „Kombination“ keine Interaktionseffekte bestehen. Im Faktor „Stufe“ treten jedoch Überschneidungen der Linienzüge für die Stufen 1–4 auf; dieser Haupteffekt kann nicht ohne Einschränkungen interpretiert werden. Ein Bonferroni-Post-Hoc-Test der Stufen über die Kombinationen hinweg ergab keine signifikanten Ergebnisse.

#### Fehler bei der Hauptaufgabe

**Figure 7:**
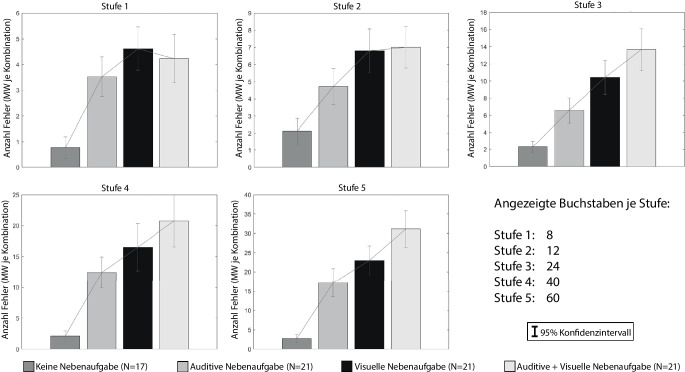

Abb. 7 stellt die Mittelwerte der Fehler dar, die von den Versuchspersonen bei der Bearbeitung der Hauptaufgabe gemacht wurden.

Unter dem Begriff „Fehler“ werden dabei sowohl falsch ausgewählte Buchstaben (z. B. „W“ angezeigt, „A“ ausgewählt), als auch übersprungene Buchstaben (keine Reaktion auf Stimulus) zusammengefasst (analog auch in Abschn. 4.2.5 und 4.2.6). Aufgrund der Beschaffenheit der Hauptaufgabe ergibt sich, dass Fehler nicht zwischen den Frequenzstufen verglichen werden können: In jeder Stufe werden unterschiedlich viele Buchstaben angezeigt, bspw. in Stufe 1 acht und in Stufe 5 sechzig Stück. Ein Fehler in Stufe 1 kann nicht mit einem Fehler in Stufe 5 gleichgesetzt werden, weil in Stufe 5 insgesamt mehr Stimuli angezeigt werden und so ein größeres Fehlerpotential besteht.

Aus Sicht der Autoren ist auch die Bildung eines Fehlerquotienten (gemachte Fehler/angezeigt Buchstaben), aus obengenannten Gründen nicht zielführend. Stattdessen werden die Stufen getrennt voneinander betrachtet und dabei zwischen den Aufgabenkombinationen differenziert.

In ihrem Artikel verglichen Delacre et al. (2019) dein Einfluss verschiedener Annahmenverletzungen auf die Alpha-Fehler von ANOVA, Welch-ANOVA und Brown-Forsythe Test. Die Autoren kamen zu dem Ergebnis, dass die Welch-ANOVA bei ungleichen Stichprobengrößen und Varianzheterogenität im Vergleich die zuverlässigsten Ergebnisse liefert und sich die Alpha-Fehlerrate innerhalb der von Bradley (1978) definierten Grenzwerte bewegt. Aufgrund von vorliegender Varianzheterogenität und der unterschiedlichen Stichprobengröße werden die Ergebnisse der Studien 1 und 3 in einer einfaktoriellen Welch ANOVA kombiniert untersucht. Die Haupteffekte und alle signifikanten Ergebnisse der Bonferroni-Post-Hoc-Tests sind in Tab. 7 dargestellt.

**Table 7:** 

**Stufe 1 Haupteffekt: **F (3, 41,90) = 27,192, *p* < 0,001, partielles η^2^ = 0,385			
**Sample 1**	**Sample 2**	**Mittelwertdifferenz**	**Sig.**
Keine NA	Auditive NA	±2,70	<0,001
Keine NA	Visuelle NA	±3,80	<0,001
Keine NA	Auditive + Visuelle NA	±3,42	<0,001
**Stufe 2 Haupteffekt: **F (3, 42,08) = 14,995, *p* < 0,001, partielles η^2^ = 0,367			
**Sample 1**	**Sample 2**	**Mittelwertdifferenz**	**Sig.**
Keine NA	Auditive NA	±2,60	0,016
Keine NA	Visuelle NA	±4,70	<0,001
Keine NA	Auditive + Visuelle NA	±4,88	<0,001
Auditive NA	Auditive + Visuelle NA	±2,29	0,030
**Stufe 3 Haupteffekt: **F (3, 40,20) = 28,219, *p* < 0,001, partielles η^2^ = 0,504			
**Sample 1**	**Sample 2**	**Mittelwertdifferenz**	**Sig.**
Keine NA	Auditive NA	±4,28	0,016
Keine NA	Visuelle NA	±8,14	<0,001
Keine NA	Auditive + Visuelle NA	±11,37	<0,001
Auditive NA	Visuelle NA	±3,86	0,024
Auditive NA	Auditive + Visuelle NA	±7,10	<0,001
**Stufe 4 Haupteffekt: **F (3, 37,76) = 46,696, *p* < 0,001, partielles η^2^ = 0,452			
**Sample 1**	**Sample 2**	**Mittelwertdifferenz**	**Sig.**
Keine NA	Auditive NA	±10,31	<0,001
Keine NA	Visuelle NA	±14,41	<0,001
Keine NA	Auditive + Visuelle NA	±18,64	<0,001
Auditive NA	Auditive + Visuelle NA	±8,33	0,003
**Stufe 5 Haupteffekt: **F (3, 37,56) = 60,756, *p* < 0,001, partielles η^2^ = 0,595			
**Sample 1**	**Sample 2**	**Mittelwertdifferenz**	**Sig.**
Keine NA	Auditive NA	±14,43	<0,001
Keine NA	Visuelle NA	±20,24	<0,001
Keine NA	Auditive + Visuelle NA	±28,36	<0,001
Auditive NA	Auditive + Visuelle NA	±13,95	<0,001
Visuelle NA	Auditive + Visuelle NA	±8,14	0,015

#### Fehler bei der visuellen Nebenaufgabe

**Figure 8:**
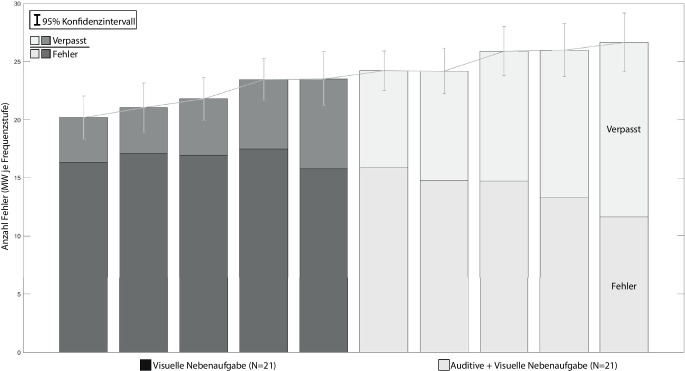

Die Mittelwerte der Fehler der visuellen Nebenaufgabe sind in Abb. 8 in einem gestapelten Balkendiagramm dargestellt. Die unteren Balkenteile repräsentieren dabei die falsch identifizierten Formenpaare. Die oberen Balkensegmente zeigen die verpassten Formenpaare, also keine Eingabe der Versuchspersonen.

Eine zweifaktorielle ANOVA mit Messwiederholung zeigt sowohl einen signifikanten Unterschied mit starkem Effekt für die Fehlerrate zwischen den Aufgabenkombinationen *HA* *+* *visuelle NA* und *HA* *+* *auditive* *+* *visuelle NA* (*F* (1,20) = 45,808, *p* < 0,001, partielles η^2^ = 0,696) als auch einen signifikanten Unterschied mit geringem Effekt für die Frequenzstufen (*F* (4,80) = 4,559, *p* = 0,002, partielles η^2^ = 0,187). Zwischen der Aufgabenkombination und den Stufen bestehen keine Interaktionseffekte. Zwischen den Frequenzstufen konnten post-hoc keine Unterschiede in den Fehlerraten der visuellen Nebenaufgabe gefunden werden.

#### Fehler bei der auditiven Nebenaufgabe

**Figure 9:**
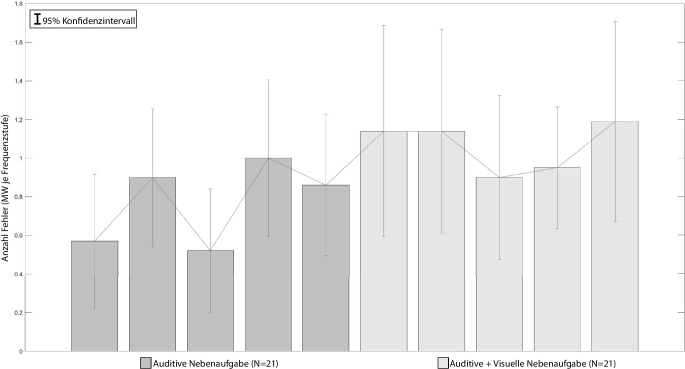

Die Mittelwerte der Fehler der auditiven Nebenaufgabe sind in Abb. 9 dargestellt. Eine zweifaktorielle ANOVA mit Messwiederholung zeigte signifikante Unterschiede zwischen den Fehlerraten der Kombinationen *HA* *+* *auditive NA* und *HA* *+* *auditive* *+* *visuelle NA* mit geringer Effektstärke (*F* (1,20) = 7,427, *p* = 0,013, partielles η^2^ = 0,271). Zwischen den Stufen über die Kombinationen hinweg konnte kein signifikanter Unterschied festgestellt werden (*F* (4,80) = 1,602, *p* = 0,182, partielles η^2^ = 0,074). Gleichermaßen bestehen keine Interaktionseffekte zwischen den Aufgabenkombinationen und Frequenzstufen.

## Diskussion

Nachfolgend werden die in Abschn. 2 formulierten Hypothesen auf ihre Gültigkeit geprüft, eine neue Experimentalaufgabe erarbeitet, die Limitationen dieser Arbeit beschrieben und ein Ausblick auf zukünftige Forschungsarbeiten gegeben.

### Bewertung der Hypothesen

Die Hypothese 1 einer ansteigenden psychischen Belastung bei steigender Aufgabenfrequenz kann teilweise bestätigt werden. Studie 1 ergab keine signifikanten Unterschiede der subjektiven Bewertung über die fünf Frequenzstufen. In Studie 3 konnte ein Unterschied nur dann nachgewiesen werden, wenn mindestens eine Stufe zwischen den Paaren liegt, mit Ausnahme zwischen Stufe 4 und 5. Sowohl in Studie 1 als auch in Studie 3 unterscheiden sich die Reaktionszeiten der Stufen signifikant voneinander. Dabei fällt die Reaktionszeit mit sinkender Buchstabenfrequenz. Dieser Effekt kann teilweise anhand De Waards (1996) *n*-Förmigen Beanspruchungs-Leistungsverlauf erklärt werden: Zu geringe Beanspruchung einer Versuchsperson (Unterforderung) kann zu Monotonie, Konzentrationsverlust und damit zu Leistungseinbußen führen. Eine höhere Buchstabenfrequenz fördert die Konzentration, erleichtert das Erkennen von Zeit-Mustern (die Buchstaben erscheinen weniger „überraschend“) und führt zur Leistungssteigerung. Die Fehlerrate lässt keine Schlüsse auf Unterschiede zwischen den Stufen zu (vgl. Abschn. 4.2.4). Zusammenfassend lässt sich sagen, dass es Unterschiede zwischen den Stufen gibt, die induzierte psychische Beanspruchung von Stufe 1 bis 5 ansteigt, jedoch keine Unterschiede in aneinander angrenzenden Stufen nachgewiesen werden konnten.

Hypothese 2 einer ansteigenden psychischen Belastung bei hinzukommenden Nebenaufgaben kann durch einen signifikanten Haupteffekt bei dem Vergleich der RSME-Bewertungen aus Studie 3 in Abschn. 4.1 als erfüllt betrachtet werden. Die Analyse der Fehlerraten bestärkt diese Erkenntnis: Wird eine Nebenaufgabe hinzugefügt, steigt die Fehlerrate in der Hauptaufgabe signifikant an (vgl. Abschn. 4.2.4).

Hypothese 3 vermutete die geringste Steigerung der psychischen Belastung durch die auditive Nebenaufgabe, eine höhere durch die visuelle Nebenaufgabe und eine sehr große durch die Kombination von visueller und auditiver Nebenaufgabe. Die Ergebnisse der subjektiven Beanspruchungsbewertung aus Studie 3 zeigen, dass sich die Kombinationen signifikant voneinander unterscheiden. Obwohl in einem Post-Hoc-Test kein signifikanter Unterschied zwischen den Kombinationen *HA* *+* *visuelle NA* und *HA* *+* *auditive NA* festgestellt werden konnte, unterstützen die Mittelwertdifferenzen die erwartete Reihenfolge aus Hypothese 3. Für die Hauptaufgabe erhöht sich die durchschnittliche Reaktionszeit je Kombination erwartungsgemäß in der Reihenfolge *HA* *+* *auditive NA, HA* *+* *visuelle NA* bis *HA* *+* *auditive NA* *+* *visuelle NA*. Analog steigt die Reaktionszeit der auditiven Nebenaufgabe, wenn zeitgleich die Hauptaufgabe und die visuelle Nebenaufgabe präsentiert werden. Die Analyse der Fehlerraten und Reaktionszeiten konsolidiert diese Einschätzung: mit Ausnahme von Stufe 1 steigen die Fehleranzahl in der Hauptaufgabe und die Reaktionszeit der auditiven Nebenaufgabe wie erwartet an. Die Kombination beider Nebenaufgaben führt dabei zu den meisten Fehlern und zur längsten Reaktionszeit. Zusammenfassend kann Hypothese 3 angenommen werden.

### Konzept der Experimentalaufgabe

Eine Experimentalaufgabe, die ein breites Beanspruchungsspektrum in Versuchspersonen zuverlässig und reproduzierbar induzieren kann, muss so entwickelt werden, dass sich aufeinanderfolgende Teilaufgaben in der induzierten Beanspruchung signifikant unterscheiden. Ansonsten kann ein physiologisches Messsystem keinen Unterschied detektieren. Aus den Ergebnissen der drei Studien und den Anforderungen, die Belastung gezielt zu variieren und Wiedererkennungseffekte zu minimieren (vgl. Städtler 1998), lassen sich die folgenden drei Bedingungen an die Reihenfolge der Aufgabenkombinationen ableiten:

1. Aufeinanderfolgende Teilaufgaben sollen sich in der Buchstabenfrequenz unterschieden.
2. Dieselbe Teilaufgabe soll in der Experimentalaufgabe nicht wiederholt werden.
3. Aufeinanderfolgende Teilaufgaben sollen sich in der Nebenaufgabe unterschieden.

**Figure 10:**
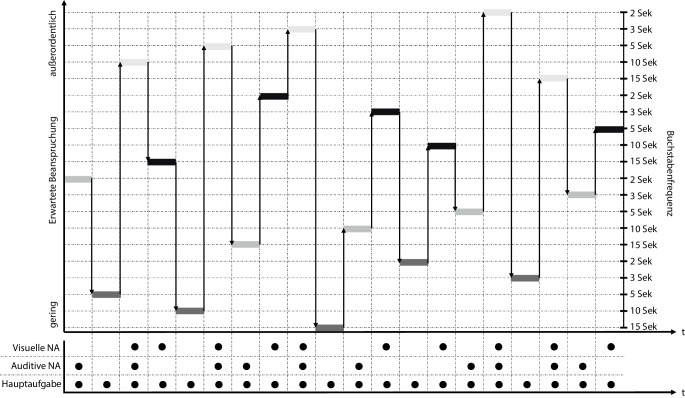

Daraus ergibt sich die in Abb. 10 dargestellte Teilaufgabenreihenfolge, die schließlich als eine zusammenhängende Experimentalaufgabe mit Übungsdurchgängen für die Haupt- und Nebenaufgaben und mit Zeitfenstern für Baseline-Messungen (relevant für z. B. Herzfrequenzanalysen) in Python (2021) implementiert wurde.

Im Programmablauf erscheint auf dem Computermonitor nach jeder Teilaufgabe (vgl. Abb. 10) ein Fenster, in dem die Versuchspersonen ihre subjektiv empfundene Beanspruchung anhand der RSME bewerten können. Diese Daten lassen sich in Kombination mit physiologischen Prädiktoren als Antwortvariablen für die Modellbildung verwenden (vgl. Abschn. 2). Durch eine solche Datenerhebung werden individuelle Unterschiede zwischen den Personen berücksichtigt. So ist es etwa denkbar, dass eine Person, die Deutsch als Fremdsprache spricht, die auditive Nebenaufgabe aufgrund der höheren kognitiven Beanspruchung durch die Fremdsprache (vgl. z. B. Costa 2020) beanspruchender empfindet als die visuelle Nebenaufgabe. Die entsprechend höhere Bewertung und die korrespondierenden physiologischen Reaktionen werden trotz der Abweichung zum erwarteten Beanspruchungszustand korrekt zugeordnet.

### Limitationen und Ausblick

Einschränkungen bei der Interpretation der Ergebnisse sind die geringen Stichprobengrößen (N1 = 17, N2 = 8, N3 = 21) und die Vergleichbarkeit der Studien 1, 2 und 3 untereinander.

Oberfeld und Franke (2013) untersuchten in ihrem Artikel den Einfluss verschiedener Stichprobengrößen (*N* = 3 bis 100), Faktorstufen (K = 4, 8, 16) und Verteilungsformen (normal, nicht normal) auf den Alpha-Fehler univarianter und multivarianter Testverfahren, unter anderem auch für die hier verwendete messwiederholte ANOVA mit Greenhouse-Geisser Korrektur. Die Autoren zeigten, dass sich der Alpha-Fehler, unter Annahme einer Normalverteilung, ab einer Stichprobengröße von *N* = 8 bei K = 4 und *N* = 16 bei K = 16 in dem von Bradley (1978) definierten Intervall für akzeptable Alpha-Fehler-Abweichungen bewegt.

Stiger et al. (1998) untersuchten in ihrem Artikel nicht nur den Einfluss kleinerer Stichproben (*N* = 20), sondern auch den Effekt einer ordinalskalierten abhängigen Variable auf u. a. messwiederholte ANOVA mit und ohne Greenhouse-Geisser Korrektur. Unter Annahme einer Normalverteilung kommen die Autoren auch hier zu dem Schluss, dass eine messwiederholte ANOVA mit kleineren Stichproben keine übermäßige Abweichung zum erwarteten Alpha-Fehler aufweist.

Aufgrund limitierender Maßnahmen zur Kontaktbeschränkung und längerer Schließungen der Laborräume durch die Corona-Pandemie konnten weniger Versuchspersonen rekrutiert und getestet werden als üblich. Mit *N* = 17, *N* = 21 und maximal 2 Faktoren (5 Stufen und 3 Aufgabenkombinationen) wird nach Oberfeld und Franke (2013) und Stiger et al. (1998) keine relevante Beeinträchtigung der ANOVA durch die Stichprobengröße in dieser Arbeit erwartet.

Die Studien 1, 2 und 3 unterscheiden sich nicht im Aufbau der Hauptaufgabe (Frequenzen, Bearbeitungszeit der Kombinationen und Evaluation), fanden jedoch in unterschiedlichen Zeiträumen und mit leicht abweichenden Randbedingungen statt, wie z. B. einer gegenüber den Studien 1 und 2 modifizierten Übungsphase der Studie 3. Aus unserer Sicht ist eine Vergleichbarkeit der 3 Studien grundsätzlich gegeben. Im Zuge der Anwendung der hier entwickelten Experimentalaufgabe in bevorstehenden Experimenten wird die Validität der Ergebnisse fortlaufend weiter überprüft werden.

Trotz der beschriebenen Einschränkungen können die Hypothesen 1 und 3 mit Vorbehalt und die Hypothese 2 vollständig angenommen werden. Durch die strategische Kombination von Frequenzstufen mit Nebenaufgaben unter zusätzlicher Variation ihrer Reihenfolge konnte eine Experimentalaufgabe entwickelt werden, die 20 erhebliche Belastungswechsel mit einem Spektrum von geringer bis sehr hoher Beanspruchung (vgl. Abb. 7) in Versuchspersonen induziert. Aufbauend auf diesen Ergebnissen soll in zukünftigen Studien ein System zur Messung psychischer Beanspruchung durch physiologische Indikatoren entworfen und erprobt werden.
